# Heparan Sulfate Proteoglycan Signaling in Tumor Microenvironment

**DOI:** 10.3390/ijms21186588

**Published:** 2020-09-09

**Authors:** Valeria De Pasquale, Luigi Michele Pavone

**Affiliations:** 1 Department of Veterinary Medicine and Animal Production, University of Naples Federico II, 80137 Naples, Italy; 2 Department of Molecular Medicine and Medical Biotechnology, Medical School, University of Naples Federico II, 80131 Naples, Italy; luigimichele.pavone@unina.it

**Keywords:** tumor microenvironment, extracellular matrix, heparan sulfate proteoglycans, remodeling, signaling

## Abstract

In the last few decades, heparan sulfate (HS) proteoglycans (HSPGs) have been an intriguing subject of study for their complex structural characteristics, their finely regulated biosynthetic machinery, and the wide range of functions they perform in living organisms from development to adulthood. From these studies, key roles of HSPGs in tumor initiation and progression have emerged, so that they are currently being explored as potential biomarkers and therapeutic targets for cancers. The multifaceted nature of HSPG structure/activity translates in their capacity to act either as inhibitors or promoters of tumor growth and invasion depending on the tumor type. Deregulation of HSPGs resulting in malignancy may be due to either their abnormal expression levels or changes in their structure and functions as a result of the altered activity of their biosynthetic or remodeling enzymes. Indeed, in the tumor microenvironment, HSPGs undergo structural alterations, through the shedding of proteoglycan ectodomain from the cell surface or the fragmentation and/or desulfation of HS chains, affecting HSPG function with significant impact on the molecular interactions between cancer cells and their microenvironment, and tumor cell behavior. Here, we overview the structural and functional features of HSPGs and their signaling in the tumor environment which contributes to tumorigenesis and cancer progression.

## 1. Introduction

The tumor microenvironment consists of a heterogeneous population of cells such as proliferating tumor cells and infiltrating inflammatory cells, the tumor stroma, blood vessels, secreted factors, and extracellular matrix (ECM) components, all together contributing to cancer development and progression. Complex and dynamic interactions between tumor cells and their microenvironment, involving cell-cell and cell-ECM contacts and the activity of soluble factors that enable these contacts, are essential to promote tumor growth, invasion, and metastasis [1,2,3]. Hence, due to the compelling role of tumor microenvironment in carcinogenesis, therapeutic strategies targeting tumor microenvironment components that interfere with the complex crosstalk between tumor cells, host cells, and their surrounding ECM are being explored [4,5,6].

The ECM constituents form a highly dynamic network that plays both structural and functional roles of key importance for development and tissue homeostasis. The composition of ECM may differ among tissues and continuously undergo remodeling both in physiological and pathological conditions [7,8,9]. The main ECM components include fibrillar proteins such as collagen, elastin, fibronectin, and laminins, glycosaminoglycans (GAGs), proteoglycans (PGs), and other glycoproteins. The interaction between ECM components and cell surface receptors and/or matrix effectors activates signal transduction cascades underlying cell differentiation, proliferation, survival, adhesion, migration, and other biological processes relevant to cancer biology [8].

Among ECM components, heparan sulfate (HS) proteoglycans (HSPGs) emerged as critical determinants in ECM assembly and functions both in health and disease [10,11]. The ubiquitously expressed HSPGs comprise diverse families of HS chains bearing protein cores that include syndecans, glypicans, perlecan, agrin, and collagen type XVIII. While perlecan, agrin, and collagen type XVIII are directly secreted in the ECM once synthesized, the transmembrane syndecans and glycosylphosphatidylinositol-anchored (GPI)-anchored glypicans are cell surface-bound HSGPs, but they can be cleaved by proteinases or heparanases, and their truncated forms can be distributed in the ECM. The sulfated moieties enable HSPGs to interact with other ECM components and a variety of ligands such as morphogens, growth factors, enzymes, cytokines, chemokines, etc. [12,13,14,15]. However, not only the sulfated pattern of HS chains dictates the binding specificity of HSPGs, but their protein core can also bind ligands, and the ECM secreted HSPG types contain functionally independent ligand-binding domains [11,12,13,16]. The HSPG binding ability is essential for regulating the distribution, availability, and signaling activity of the ligands.

The main activity attributed to HSPGs is to serve as co-receptors for morphogens/growth factors, thus enhancing signaling activation of their respective receptor, however, HSPGs can act as receptors themselves and can transactivate receptors in adjacent cells [10,11,12,13,15,17]. In addition, HSPGs are involved in endocytosis and vesicular trafficking [18]. By acting as intermediaries between ECM and intracellular signaling pathways, HSPGs regulate a variety of physiological and pathological processes including tissue development, morphogenesis, cell proliferation, apoptosis, adhesion, motility, wound healing, inflammation, and tumorigenesis [10,11,17,19,20,21,22,23,24,25,26,27,28].

Altered expression and structural variability of HSPGs have been associated with an extensive remodeling of tumor microenvironment where HSPGs not only contribute to the formation of a structural framework for tumor growth but are also involved in the regulation of cell-matrix and cell-cell interactions, and cell signaling [29,30,31,32,33,34,35]. They are able to modulate cancer cell phenotype, the development of drug resistance, and tumor stroma angiogenesis [36,37,38,39,40,41]. Differential expression and structure/activity modifications of HSPGs have been found in several cancers and may correlate with either inhibitory or tumor-promoting activity. This review focuses on the structural and functional alterations of HSPGs in tumor microenvironment that have a significant impact on tumor growth and progression. We also discuss the advancements in the development of cancer therapies targeting HSPGs.

## 2. Structural Features, Biosynthesis, and Enzymatic Modification of HSPGs Regulating Cancer Promotion and Progression

The HSPGs are glycosylated proteins characterized by a core protein carrying covalently attached HS chains (Table 1). Thirteen genes encode HSPG core proteins. They include the genes encoding for cell surface-tethered 4 syndecan (SDC1-4) and 6 glypican (GPC1-6) isoforms, and 3 encoding for the basement membrane- and -ECM localized perlecan, agrin, and collagen type VIII [11]. Syndecan isoforms are transmembrane glycoproteins with the extracellular domain harboring HS chains and chondroitin sulfate chains, and highly conserved transmembrane and cytoplasmic domains which mediate multimerization and interactions with intracellular proteins, respectively. Glypicans are proteins anchored to the cell membrane by GPI, and with HS chains attached near the juxtamembrane region. Perlecan, agrin, and collagen type XVIII are localized in the ECM, including the basement membrane zone [11,16,42].

In HSPGs, the HS chains are constituted by a long unbranched backbone of disaccharide units of D-glucosamine and uronic acid (D-glucuronic and L-uronic acids) carrying negatively charged carboxylated or N- and O-sulfated groups generated through tightly regulated post-translational reactions in the Golgi apparatus upon the arrival of the core protein from the endoplasmic reticulum [17]. The HSPG biosynthetic process starts with the attachment of a specific serine residue of the core protein to a tetrasaccharide linker (glucuronic acid-galactose-galactose-xylose) bearing HS chains; this reaction is catalyzed by xylosyltransferase (XTLY). Exostosin (EXT) enzymes catalyze the elongation of HS chains through the alternate addition of glucuronic acid and N-acetylglucosamine. Then, the HS backbone undergoes modifications involving N-deacetylation and N-sulfation of glucosamine, C-5 epimerization of glucuronic acid to iduronic acid, 2-O-sulfation and 3-O-sulfation of uronic acid and glucosamine, respectively, and 6-O-sulfation of N-acetylated or N-sulfated glucosamine residues. Additional modifications occur at the cell surface or ECM through the action of 6-O-endo-sulfatases and/or the endoglycosidase heparanase. The controlled biosynthesis and post-synthetic modifications of HS chains provide an enormous potential of heterogeneity and structural variability of HS chains which accounts for a wide variety of HSPG interactions with regulatory proteins and, in turn, for their biological activities [12,13,14,15,43]. Several studies have demonstrated that there are cell and tissue-specific changes in the HS chain synthetic pathway in cancer cells and tissues in vitro and in vivo, thus suggesting a close involvement of HS chain biosynthetic machinery in carcinogenesis [30,44,45]. These changes may concern either the expression and/or activity of HS synthetic and modifying enzymes, or changes in the HSPGs protein core.

The genetic loss of *NDST4*, a member of the N-deacetylase/N-sulfotransferase (NDST) family, correlates with an advanced pathological stage and poor survival in colorectal carcinomas [46]. Interestingly, depending on the metastatic nature of the tumor and its localization, differential expression in the transcription of genes involved in the epimerization and sulfation of uronic acid, and glucosamine sulfation were detected in left- and right-sided colorectal cancers [31]. Defective HS-3-O-sulfation due to methylation-associated repression of HS glucosamine 3-O-sulfotransferase gene (*3-OST*) results in being associated with chondrosarcoma progression [47], whereas hypermethylation of the *3-OST* gene is associated with poor survival in non-small cell lung cancer [48]. In addition, HS-2-O-sulfotransferase (2-OST) results in being essential for the proliferation and invasion of prostate cancer cells [49]. Overexpression of HS glucosamine 6-*O*-sulfotransferase-2 (*6-OST*) has been reported in colorectal cancer and gastric cancer, while it results in being downregulated in glioma [50,51,52].

Mutations in *EXT1* or *EXT2*, members of the EXT family of glycosyltransferases are responsible for hereditary multiple osteochondromas that may degenerate into chondro- or osteo-sarcomas [53]. Furthermore, mutations in *EXT2* have been detected in breast tumor patients, and thyroid cancer [54,55,56]. Epigenetic inactivation of *EXT1* by promoter hyper-methylation preventing HS chain synthesis is observed in leukemia and non-melanoma skin cancer [57,58]. An antiproliferative effect of D-glucuronyl C5-epimerase (GLCE) has been ascertained in breast and small lung cancer cells [59,60,61], whereas increased GLCE expression has been associated with advanced stages of prostate tumors [62,63]. Although many other examples of the dysregulation of HS biosynthetic and post-synthetic modifying enzymes in carcinogenesis have been reported (Table 2), the complex changes of their expression in different cancers remains still to be explored.

In addition to the differential expression and/or activity of the enzymes involved in the biosynthesis or post-synthetic modification of HS chains, HSPG core proteins may also affect cancer development and progression, either by preventing or promoting these processes [10,11,36,39,40]. The alterations in the expression levels of HSPGs depend on their location and may represent a hallmark of the metastatic or non-metastatic nature of the tumor. For example, while SDC1 results in being overexpressed in left-sided colorectal tumors independently from the presence of metastasis, it results in being upregulated only in metastatic right-sided colorectal cancers [31,105]. However, a significant reduction of cell surface tethered SDC1 and an increase of shed SDC1 in the ECM has been observed as a function of tumor progression and aggressiveness, suggesting the involvement of post-transcriptional mechanisms in SDC1 expression in this type of tumor. Differential regulation of SDC1 expression as well as of the other SDC isoforms, GPCs, and the other HSPGs has been found in several tumors (Table 3) [105,106,107,108,109,110,111,112,113,114,115,116,117,118,119,120,121,122,123,124,125,126,127,128,129,130,131,132,133,134,135,136,137,138,139,140,141,142,143,144,145,146,147,148,149,150,151,152,153].

High levels of SDC1 have been detected in squamous cell carcinomas such as those from cervix uteri and esophagus, in invasive urothelial cancer, and lung cancer [108]. Overexpression of SDC1 correlates with tumor aggressiveness and poor survival in triple-negative breast carcinoma [109]. Both SDC1 and SDC4 are overexpressed in papillary thyroid carcinoma [118]. Conversely, reduced expression of SDC1 has been found in mesothelioma, non-small-cell lung cancer, prostate cancer, and sarcoma [35,108,110,111]. SDC2 expression is upregulated in breast, colon, and pancreatic cancers, and melanomas, whereas high levels of SDC2 in neuroendocrine tumors correlate with a better survival of patients [112,113]. On the contrary, a tumor-suppressor function for SDC2 correlated to apoptosis dysregulation in osteosarcoma has been suggested [114]. Elevated expression levels of SDC3 have been reported in bladder and ovarian cancer, and renal cell carcinoma [115,116,117], whereas low levels of SDC3, SDC4, GPC1, and GPC3 are expressed in neuroblastoma [35].

Overexpression of GPC1 is a hallmark of breast cancer, esophageal squamous cell carcinoma, and gliomas [119,120,121]. The upregulation of GPC1 and GPC4 is found in pancreatic cancer [122,123]. High expression of GPC2 has been detected in neuroblastoma and other pediatric cancers such as medulloblastoma and retinoblastoma [124,125]. While CPG3 results in being overexpressed in liver cancer, lung squamous cell carcinoma, neuroblastoma, ovarian cancer, testicular germ cell tumor, thyroid cancer, yolk sac tumor and other cancers, reduced levels of GPC3 have been found in breast cancer, colorectal cancer, mesothelioma, non-small-cell lung cancer, neuroblastoma, and renal cell carcinoma [35,105,125,126,127,128,129,130]. Overexpression of GPC4 mRNA has been detected in metastatic colorectal cancer, where GPC1, GPC3 and GPC6, perlecan, and collagen type VIII result in being downregulated [31,35,105]. While GPC5 expression is downregulated in breast cancer, glioma, hepatocellular carcinoma, lung cancer, pancreatic cancer, prostate cancer, it results in being upregulated in rhabdomyosarcoma [35,134,135,136,137,138]. Overexpression of GPC6 is associated with gastric adenocarcinoma and metastatic progression of cutaneous melanoma [140]. Increased expression levels of perlecan have been found in hepatocellular carcinoma, melanoma, pancreatic and prostate cancer, whereas the upregulation of the expression of agrin has been demonstrated in oral squamous cell carcinoma, hepatocellular carcinoma, cholangiocarcinoma, lung carcinoma, oral squamous cell carcinoma, and rectal cancer [35,38,144,145,146,148,149,150,151,152]. Reduced levels of perlecan correlate with the progression of breast cancer, colorectal cancer, lung cancer, ovarian cancer, and fibrosarcoma [35,38,105,143,144,147]. Finally, type VIII collagen results in being elevated in melanoma, lung, breast, ovary, prostate, and pancreatic cancers [35,38,153].

Noticeably, in some cases, the HS chain and the protein core of an HSPG may have a distinct impact on the same tumor. For example, in Lewis lung carcinoma, clones with a low metastatic potential contain high levels of SDC2, whereas, in highly metastatic clones, SDC2 overexpression reduces the invasive potential of cells due to the binding of HS chains to the fibronectin [112]. The expression patterns of HSPGs in tumor cells and microenvironment in some cases correlate with those of ligands that require HSPGs to elicit their cellular responses. [33,34,35,36,38,39,40,41,106,107]. The aberrant expression of specific HSPGs in the various types of cancers significantly affects HSPG-ligand binding and subsequent signaling, thus determining the malignancy of the tumor phenotype. Therefore, HSPGs can serve as cancer-type-specific biomarkers, prognostic factors, and therapeutic targets.

It has been well established that cell surface and ECM secreted HSPGs may undergo a cleavage process known as “shedding” which regulates the amount of HSPGs tethered to the cell surface and that present in the pericellular microenvironment [10,11,12,13,14]. The enzymes involved in the HSPG shedding depend on the type of HSPG and include the endoglycosidase heparanase and endosulfatases that modify the structure of HS chains; matrix metalloproteinases (MMPs) and ADAMs, composed of a disintegrin and MMP proteases, for SDCs shedding; the extracellular lipase Notum that cleaves the GPI anchor of GPCs; and other proteases that cleave the core proteins of ECM secreted HSPGs [33,34,37,39,42,154,155]. The cleaved HSPG products released in the tumor microenvironment may have a significant impact on cancer cell behavior [91]. The proteolysis of the SDC juxtamembrane region releases their whole ectodomains in the ECM [29]. Soluble SDC1 promotes the growth of myeloma tumors in vivo, while shed SDC2 enhances colon, lung, and breast cancer progression [11,91,100,101,156,157]. SDC-1 shedding is associated with increased mitogenic activity and invasive potential of pancreatic cancer cells, whereas shedding of SDC4 in human endothelial cells promotes wound healing, angiogenesis, and inflammation [156,157]. Furthermore, SDC1 shedding has been shown to trigger a switch from a proliferative to an invasive phenotype of breast cancer cells [158]. The cleavage of GPC1 by ADAM17 plays a role in the adhesion, proliferation and migration of oral squamous cell carcinoma cells [159]. At the basement membrane of the cells, perlecan can undergo shedding through heparanase, MMPs, and other proteases [145]. The C-terminal fragment of perlecan, known as endorepellin, resulting from the proteolytic cleavage of perlecan, may undergo further proteolysis that leads to the release of the C-terminal endorepellin fragment LG3 whose levels are reduced in breast cancer [160]. LG3 and other endorepellin fragments have been found in the secretome of colon and pancreatic cancers [161,162]. On the other hand, the proteases cathepsin L and elastase cleave the N-terminal hinge domain of collagen type VIII, releasing the 22-kDa fragment endostatin which is known to inhibit the progression of several types of malignant tumors, including melanomas, fibrosarcomas, and hemangioendothelioma [163,164]. Both MMPs and the serine protease cleave the HSPG agrin giving rise to 100-, 90-, and 22-kDa fragments which are involved in cancer growth [38].

The above reported are only few examples of the broad impact of HSPG structural features in cancer development and progression. Interestingly, the complexity of structural properties of HSPGs translates in a variety of biological activities that may either positively or negatively regulate tumor initiation and progression.

## 3. Functional Properties of HSPGs in Tumor Microenvironment

**Figure 1:**
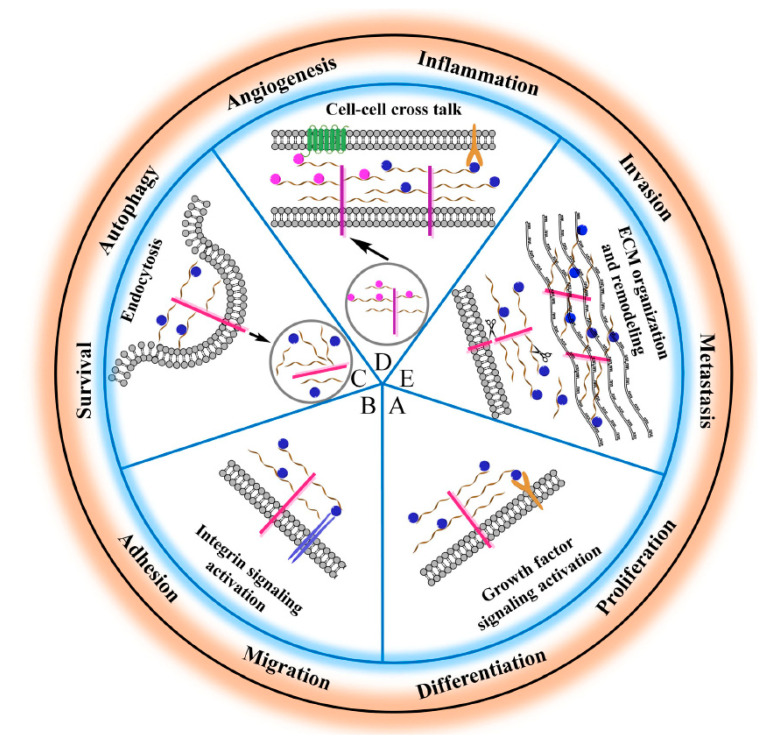
Schematic representation of the main HSPG functions relevant to cancer cell biology. (**A*,*D**) HSPGs serve as a signaling co-receptor for growth factor activity, allowing a proper presentation of them to their cognate receptors, on the same or adjacent cells. In panel D, transcellular transport of a ligand (i.e., chemokine) bound to HS chains and its presentation at the cell surface is also shown. (**B**,**D**) HGPGs bind integrins modulating their downstream signaling that regulates cytoskeleton organization as well as cell adhesion, spreading and sensing mechanical stress. (**C**) HSPGs act as endocytic receptors and undergo constitutive as well as ligand-induced endocytosis: exosomes, cell-penetrating peptides, polycation–nucleic acid complexes, lipoproteins, growth factors, and morphogens enter cells through this mechanism. Internalized cargo can be sorted for lysosomal degradation, escape into the cytosol, or recycle back to the plasma membrane. (**E**) HSPGs are critical determinants of extracellular matrix (ECM) assembly and remodeling. If the HSPGs perlecan, agrin, and collagen type XVIII are directly secreted in the ECM, cell surface-tethered HSPGs (syndecans and glypicans) undergo proteolytic cleavage of their ectodomains or to cleavage of HS chains by heparanases and their truncated forms can be distributed in the ECM. Here, HSPGs act as a reservoir of growth factors and supply them to target cells when needed. Otherwise, they may act as a barrier for growth factors, by preventing their passive diffusion over longer distances, instead of confining them to the vicinity of producing cells. Overall, HSPGs control fundamental cellular processes (i.e., cell adhesion, migration, etc.) whose dysregulation underlies tumor development and progression.

The sulfated HS side chains bearing multiple negative charges, but also protein cores, allow HSPGs to bind and interact with a broad variety of signaling effectors in the tumor microenvironment [165]. These HSPG-ligand interactions serve multiple functions including the modulation of ligand distribution and function, the restriction of ligand range of action on target cells, the prevention of ligand degradation, the generation of morphogen gradients, the proper presentation of growth factors to their cognate receptors, the transactivation of receptors in adjacent cells, the promotion of endocytosis and vesicular trafficking, etc. [7,8,10,11,12,13,14,15,17,18]. In addition to a well-established role in development [20,23,26,104,165,166,167], HSPG-ligand interactions play major roles in tumor stroma and tumor microenvironment by regulating cellular proliferation, differentiation, adhesion, migration, apoptosis, angiogenesis, inflammation, invasion, and metastasis [3,22,24,25,28,33,34,35,36,37,38,39,40,107,143,165,168] (Figure 1).

### 3.1. HSPG-Regulated Mechanisms in Cell-Matrix and Cell-Cell Interactions

One of the most studied molecular mechanisms of ligand-receptor complex formation and signaling activation mediated by HSPGs is related to the action of fibroblast growth factor (FGF) family members and their tyrosine kinase receptors (FGFR) [10,11,12,13,14,15,169]. The HS chain of HSPGs binds the FGF ligand and receptor forming a ternary complex that promotes FGFR dimerization, and in turn activates signaling. Depending on the tumor type, HSPG-regulated FGF binding and receptor dimerization triggers the activation of four main signaling pathways, including mitogen-activated protein kinase (MAPK)/extracellular signal-regulated kinase (ERK), phosphatidylinositol 3-kinase (PI3K)/protein kinase B (AKT), Janus kinase (JAK)/signal transducer and activator of transcription (STAT), and protein kinase C (PKC) pathways [15,35,125,170]. However, other HSPG-mediated FGF/FGFR downstream signaling, such as Jun N-terminal kinase (JNK), ribosomal protein S6 kinase 2 (RSK2), and Rho GTPase pathways, have been described to play a role in some cancers [35,125,171,172,173].

Commonly, the MAPK/ERK signaling cascade activated by FGFs is implicated in cell growth and differentiation, the PI3K/AKT signaling cascade in cell survival and cell fate determination, and PKC in cell polarity [174]. For example, these pathways are involved in SDC1 activation of FGF2-FGFR1 complex formation and downstream signaling leading to malignant transformation in lymphomas, breast, and prostate cancer [16,106,107,175,176]. However, in breast cancer, while membrane-bound SDC1 promotes cell proliferation and inhibits invasion through FGF2 mediated MAPK signaling, soluble SDC1 deriving from proteolytic cleavage of membrane-bound SDC1 may trigger a switch from a proliferative to an invasive phenotype through Rho GTPase pathways [159]. The shedding of SDC1 serves an important role in the regulation of FGF2 signaling activation of the PI3K/Akt pathway that promotes epithelial-mesenchymal transition, invasion, and metastasis of pancreatic cancer cells [177]. In gliomas, GPC1 contributes to enhance mitogenic signaling via forming a ternary complex with FGF2 and the FGFR and activating both MAPK/ERK and PI3K/AKT pathways [178,179]. In rhabdomyosarcomas, GPC5 enhances FGF2 signaling that leads to mesodermal cell proliferation without inducing myogenic differentiation [134]. Furthermore, GPC5 regulates lung cancer development through a complex pathway network, including FGF-mediated activation of MAPK, PI3K, and STAT pathways [180]. The HS chains of perlecan are known to bind FGF2 promoting receptor activation, and mitogenic and pro-angiogenic signaling in different tumors, whereas the protein core of perlecan is implicated in FGF7 binding and activation of its receptor and downstream MAPK signaling leading to human colon carcinoma cell growth [38,146,181].

**Figure 2:**
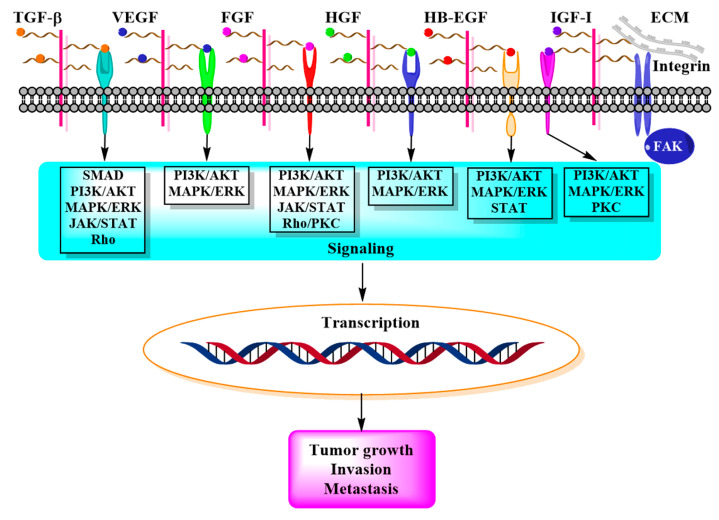
Schematic representation of the interaction between HSPGs, growth factors, and receptors, and main downstream signaling pathways that lead to tumor development and progression.

In addition to FGF, HSPGs bind several other growth factors such as hepatocyte growth factor (HGF), epidermal growth factor (EGF), heparin-binding epidermal growth factor-like growth factor (HB-EGF), transforming growth factor (TGF) beta, vascular endothelial growth factor (VEGF), and insulin-like growth factor-1 receptor (IGF1R), and modulate their signaling in a context-dependent fashion [13,15] (Figure 2).

The HSPG-mediated signaling activation of HGF released in the tumor microenvironment and of its receptor c-MET promotes ECM remodeling, inflammation, migration, angiogenesis, and invasion [182,183,184]. For example, in myeloma, shed SDC1 promotes HGF paracrine signaling that involves MAPK and PI3K cascade activation resulting in enhanced cell proliferation and survival [176,185,186]. In pancreatic cancer, HSPG-mediated activation of HGF/c-MET signaling induces proliferation and migration of tumor cells through the activation of ERK1/2 but not the AKT pathway [187]. Dysregulation of HSPG-regulated HGF/c-MET signaling in tumor microenvironment plays a key role in hepatocarcinoma [188]. Strong evidence demonstrates a role for loss of HB-EGF in the tumor microenvironment in neuroblastoma pathogenesis [189]. Indeed, HSPG-mediated binding of soluble HB-EGF with EGF receptor activates ERK1/2 and STAT3 signaling pathways, resulting in neuroblast differentiation and decreased proliferation [189]. Both SDC4 and GPC1 play a role in the EGF receptor signaling activation involving PI3K/AKT, MAPK/ERK, and JAK/STAT pathways that affect the proliferative, invasive, and migratory abilities of colon cancer cells [190]. Furthermore, SDC1 affects AKT and STAT3 signaling pathways activated by the EGF receptor in breast cancer stem cells from triple-negative breast cancer [191]. On the other hand, the HS chains of shed SCD1 bind HB-EGF, and thereby activate MAPK/ERK downstream signaling in colorectal cancer [177].

The shedding of HS chains from SDC1 in hepatocarcinoma cells facilitates lymphatic endothelial cell proliferation through VEGF-C induced ERK signaling pathway [98]. In myeloma, SDC1-mediated activation of the VEGF receptor on adjacent endothelial cells promotes AKT and ERK signaling and stimulates tumor angiogenesis [192]. Similar VEGF activation by SDC1 occurs in melanoma and ovarian carcinoma [193]. In pathologic lymphangiogenesis, association between SDC4, VEGF-C, and VEGF receptor-3 triggers activation of ERK and AKT pathways leading to mitogenic and survival responses [194]. The binding of shed perlecan to VEGF promotes activation of VEGF2 receptor signaling thus sustaining cell survival via the AKT pathway and tumor angiogenesis in hepatoblastoma [195].

In pancreatic cancer cells, GPC1 interaction with TGF-β1 promotes SMAD pathway activation resulting in cell growth inhibition [196,197]. However, TGF-β signaling may play a dual role in both pro-tumorigenic and tumor-suppressive of pancreatic cancer, depending on tumor stage and microenvironment [198]. Indeed, besides SMAD activation, TGF-β signaling can also be transduced through the non-canonical pathways that include PI3K/AKT, JNK, MAPK, and Rho GTPase pathways [199]. In glioblastoma, the stem-like population glioma-initiating cells rely on TGF-β for self-renewal, through activation of the JAK-STAT pathway [199]. In hepatocellular carcinoma, GPC3 regulates TGF-β2 signaling that involves both SMAD and MAPK/ERK pathways [200]. In fibrosarcoma, SDC2 mediates TGFβ2 transcriptional regulation via Smad signaling that affects cell adhesion [112,201]. In the same type of cancer, SDC2 also mediates IGF-I-induced activation of the ERK pathway facilitating cell migration [202]. A significant role of SDC4 on IGF-I receptor activation, together with the involvement of integrins and estrogen receptors, leading to MAPK, PI3K/AKT, and/or PKC signaling pathways, in the breast cancer cell aggressiveness has been established [203]. Furthermore, HSPG-mediated association of IGF-I with β1 integrin modulates adhesion and migration of human multiples of myeloma cells via phosphorylation of FAK and paxillin, and activation of ERK and PI3K/AKT signaling [204].

In addition to acting as co-receptors for growth factors, HSGPs provide a unique functional activity to the processes of cell-matrix and cell-cell adhesion relevant to cancer initiation and progression [40]. Indeed, HSPGs are able to bind matrix proteins such as fibronectin, laminin, thrombospondin, and collagens, and to modulate integrin activation either by direct binding or exposing the binding sites of matrix proteins for integrin engagement, thus affecting focal adhesion assembly/disassembly and intracellular signaling that regulates cell adhesion, spreading, and sensing mechanical stress [7,8,10,11,12,13,165,205,206,207]. The ectodomain and HS chains of SDC1, through αvβ3 integrin, induce ECM fiber alignment that promotes the directional migration and invasion of breast carcinoma cells [208]. A ternary complex formed by SDC1 ectodomain, IGF1 receptor, and αvβ3 integrin transduces angiogenic signals [209]. The interaction of the extracellular domain of SDC1 with αvβ3 and αvβ5 integrins regulates angiogenesis and tumorigenesis in human mammary carcinoma cells, and myeloma [192,210]. On the other hand, the interaction of the SDC1 cytoplasmic domain with the laminin receptor α6β4 integrin regulates ErbB2 tyrosine kinase activation leading to human squamous carcinoma cell spreading [211]. The protein core of SDC1 supports α2β1 integrin-mediated cell adhesion to collagen, thus negatively regulating carcinoma cell migration and invasion [111,212].

In addition to SDC1, also SDC2 acts as a co-receptor of α2β1 integrin, thus playing an important role in regulating actin cytoskeleton organization and focal adhesion kinase signaling [16,213]. Such cooperation between SDC2 and α2β1 integrin represents a possible mechanism underlying the tumorigenic activity of colon cancer cells [214]. This property correlates with the induction of differentiation toward a migratory mesenchymal phenotype of colorectal cancer-derived HT-29 M6 epithelial cells [214]. In malignant breast cancer cells, SDC2 interaction with β1 integrin promotes the invasive capacity of the cells by regulating the Rho GTPase activity [215]. SDC2 also cooperates with α5β1 integrin for regulation of actin-cytoskeletal organization in cell adhesion to fibronectin in Lewis lung carcinoma-derived metastatic cells, thus affecting their invasive capacity [216]. The integrin-dependent focal adhesion kinase (FAK) regulates SDC2 induced tumorigenic activity of HT1080 fibrosarcoma and melanoma cells [217,218]. Furthermore, SDC2 enhances FAK phosphorylation and the downstream extracellular signal-regulated kinase (ERK) activity in colon cancer cells [219]. The involvement of SDC4 interaction with β1 integrin in the development and metastasis of renal carcinomas has been demonstrated [186]. While SDC4 interaction with α6β4 integrin mediates mammary carcinoma cell migration [175], downregulation of SDC4 by FGF2-dependent dephosphorylation of FAK promotes the migration of melanoma cells [220,221]. Activation of FAK by SDC4 in epithelial tumor cells resulting in the transmission of mechano-transduction signals is important for cell spreading, actin cytoskeleton assembly, and cell contractility [222]. A ternary complex formed by SDC4, α5β1 integrin, and endothelial surface glycoprotein Thy-1 supporting cell-cell adhesion modulates mechano-signaling in melanoma cells [223]. Finally, it has been shown that α-dystroglycan and β1 integrin act as receptors for perlecan in oral precancerous lesions prior to the invasion, and the perlecan-induced signals to these receptors trigger cell differentiation and proliferation of oral carcinoma cells [224]. On the other hand, endorepellin, the C-terminal domain of perlecan, by simultaneously engaging α2β1 integrin and VEGF receptor 2 inhibits tumor angiogenesis [225]. The basal lamina and ECM localized HSPG agrin interact with αvβ1 integrin activating mechanotransduction signaling which promotes human liver cancer [149].

### 3.2. HSPG-Regulated Mechanisms in Tumor Microenvironment Remodeling

Multiple evidence demonstrates that HSPGs require proteolytic enzymes for ECM remodeling and for modulating cell signaling in tumor microenvironment. Such an interplay between proteolytic enzymes and HSPGs greatly contributes to the cancer pathogenesis [8,33,37,42,226]. In particular, the metalloproteinases MMPs, ADAMSs, ADAMS with thrombospondin motifs (ADAMTSs), and cathepsins are among the proteinases that cooperate with HSPGs in all the stages of cancer development and progression, although in a cell- and tissue-specific manner. In addition to the role of metalloproteinases in shedding which releases the ectodomain of cell surface-tethered HSPGs into the extracellular milieu with the already described impact on tumor cells, HSPGs contain docking sites for these proteases which allow the formation of complexes and their allosteric activation. Indeed, SDC2 acts as a docking receptor for pro-MMP-7 in colon cancer cells, promoting pro-MMP-7 processing into the active MMP-7, and subsequent cleavage of MMP-7 substrate E-cadherin, which, in turn, results in enhanced cell migration [219,227]. Similarly, GPCs associate with secreted MMP-9 to mediate motility of colon adenocarcinoma cells [228]. The binding of SDC4 to ADAMTSs promotes their activation, and subsequent tumorigenic signaling [229]. Furthermore, HS chains of HSPGs can simultaneously interact with an active MMP and a substrate, forming a trimeric complex [230]. For example, the binding of SDC1 to ADAMTS-4 and MMP-17 triggers the activation of ADAMTS-4 [231]. HSPGs also interact with the cathepsin family of proteases that play key roles in several human diseases, including inflammation and cancer [232,233,234,235,236,237]. In tumor microenvironment, the interaction between HS side chains of HSPGs and secreted cathepsins regulates the stability and activity of these proteases, by protecting them from alkaline pH-induced de-activation, facilitating their autocatalytic activation, and promoting conformational changes in their structure that enhance their affinity for substrates [234,236,237]. The HSPGs perlecan and collagen XVIII serve as substrates for specific cathepsins resulting in the generation of endorepellin and endostatin, respectively, whose activity in tumor microenvironment remodeling and cancer progression has been well established [163,164].

Finally, in tumor microenvironment, HSPGs are involved in compartment exchanges between cells through extracellular vesicles (EVs), thus regulating communication between malignant and stromal cells in tumor development [168]. It has been proposed that EV-associated HSPGs may function as a dynamic reservoir of signaling molecules with potential implications in the exchange of ligands between EVs and tumor target cells [238]. The release of EV within the tumor microenvironment represents a mechanism by which cell-to-cell transfer of bioactive molecules occurs with a broad impact on tumor growth, angiogenesis, and invasion [239].

In conclusion, HSPGs may regulate tumor microenvironment and cancer cell behavior through either binding growth factors or their interaction with other effectors, resulting in different types of downstream intracellular signaling that contribute to tumor promotion and progression.

## 4. Heparan Sulfate Proteoglycans as Therapeutic Targets for Cancer

Since already few years, HSPGs have been explored as potential targets for the treatment of cancers. However, due to the polyhedric nature of these molecules in terms of both structure and functions, different strategies have been developed to target HSPGs for cancer therapy. Specific domains of proteoglycan core and/or HS chains as well as HSPG synthetizing and remodeling enzymes represent potential therapeutic targets [205]. Among the explored approaches, there is the use of high-affinity antibodies recognizing functional epitopes of HSPGs, HS mimetic compounds, cationic proteins which interact with the highly anionic sulfate and carboxylate moieties of HS chains, natural and synthetic peptides, small organic molecules that may affect either HSPG-protein interactions and subsequent signaling or the HSPG biosynthetic machinery [4,5,6,29,32,37,155,156,165,239,240,241,242,243]. Some examples of HSPG targeting-based therapeutics for cancer treatment are reported in Table 4.

Several antibodies targeting distinct HSPG domains have been developed to date. An anti-GPC1 monoclonal antibody has shown potent antitumor activity in esophageal squamous cell carcinoma [244], whereas a human monoclonal antibody against GPC3, HS20, destroying Wnt3a and GPC3 interaction and subsequent signaling, exhibits elevated antitumor activity in liver cancer [245,246]. Two forms of antibody therapeutics targeting GPC2 have been successfully developed for neuroblastoma treatment [247]. The human antibody OC-46F2, specific for the ectodomain domain of SDC1, has proved to inhibit tumor growth in experimental human models of melanoma and ovarian carcinoma by blocking angiogenesis [193,248]. In some cases, antibody-drug-conjugates (ADC) consisting of a highly cytotoxic small-molecule covalently linked to a monoclonal antibody that recognizes a cell surface antigen have been developed. Indeed, a GPC2-targeted ADC obtained by conjugating a GPC2 directed antibody with pyrrolobenzodiazepine dimers resulted in being effective in neuroblastoma [249]. Furthermore, an ADC composed of an anti-GPC1 antibody conjugated with auristatin F, an anti-tubulin compound that inhibits cell division, has shown to be effective in uterine cervical squamous cell carcinoma [250].

Both saccharidic and non-saccharidic HS mimetics have shown to affect tumor cells and components of tumor microenvironment through different mechanisms, including the inhibition of cell surface-tethered HSPG signaling and HSPG-mediated cell adhesion, spreading, and angiogenesis [165,251]. Small HS mimetics molecules result in being effective in various types of cancers either administered alone or in combination regimens and are characterized by good safety and tolerability profiles [242]. A sulfated non-saccharide mimetics of heparin hexasaccharide, G2.2, inhibits colon cancer stem cells [252]. The HS mimetics OTR4120 and OTR4131 exhibit anti-tumoral effects in human hepatocellular carcinoma by interfering with HSPGs-mediated RANTES signaling [253]. Synstatin, a short peptide mimicking the SDC1 ectodomain responsible for αvβ3 or αvβ5 integrin/IGF1 complex formation and receptor activation, has been proved to be effective in mammary tumors and hepatocellular carcinoma [210,226,254]. Another approach in cancer therapy uses HS mimetics in conjunction with inhibitors of the exosites of proteases (i.e., cathepsins), thus interfering with HS/proteinase binding and proteinase catalytic activities [254].

In addition, targeting HSPG biosynthetic and post-translational modifying enzymes such as endosulfatases and heparanase represents an effective therapeutic intervention for cancer treatment [266,267,268,269,270]. An approach is represented by the manipulation of HSPG synthesis using xylosides that, competing with core proteins for HS binding, promote the secretion of xyloside-primed HS chains and core proteins with reduced, or completely lacking, HS chains [165]. The reduced glycosylation of cell surface proteoglycans affects HSPG-dependent growth factor and chemokine signaling, thus inhibiting angiogenesis, tumor growth, and invasion. Treatment with xylosides also attenuates EV-mediated intercellular transfer of signaling molecules regulated by HSPGs, resulting in a reduction of cancer cell migration and invasion [238,239]. On the other hand, different modalities for targeting EV-mediated intercellular communications have been proved to represent a useful strategy to prevent tumor progression and metastasis [271]. In addition, HS mimetics as well as antibodies, and other modulators have been developed to target heparanase and sulfatases involved in the regulation of HSPGs in tumor microenvironment [92,101,165,251,260,261,262,263,264,265,266,267,268,269,270]. Indeed, the HS mimetics PI-88, PG545, and M402 have been shown to exert anti-angiogenic and antimetastatic effects by inhibiting heparanase in several types of cancers [89,224,225,226,227,228,229,230]. Furthermore, heparanase neutralizing monoclonal antibodies attenuate myeloma and lymphoma tumor growth and dissemination [155,251,261,262,265,266]. Recently, a novel class of triazole-thiadiazole small molecules with heparanase inhibitory activity has shown the ability to reduce the metastatic potential of hepatocellular carcinoma [267]. In addition to heparanase, sulfatases that remove the O-sulfate group from HS chains have been explored as targets for cancer therapy [91]. The human sulfatase 2 (SULF2) inhibitor 2,4-disulfophenyl-*N*-tert-butylnitrone (OKN-007) exhibits antitumoral activity in hepatocellular carcinoma and glioblastoma by affecting TGFbeta1/SMAD signaling, and cell proliferation and angiogenesis, respectively [268,269]. On the other hand, proteasomal inhibitors such as MG132, Lactacystin, and Bortezomib treatment abolish SULF2 expression in multiple breast cancer cell lines [270]. Inhibition of human sulfatase 1 (SULF1) inhibits the malignant phenotype of gallbladder carcinoma cells by hindering the cell response to growth factors [272]. Thus, the modulation of tumor microenvironment by affecting the structure and/or activity of HSPGs represents an effective therapeutic strategy for preventing tumor growth and progression.

## 5. Concluding Remarks

A huge amount of data demonstrates that HSPGs are key players in tumor growth, invasion, and metastasis, due to their capability to influence tumor microenvironment and, in turn, tumor cell fate. Indeed, these multifunctional molecules by interacting with matrix effectors, cell surface receptors, and enzymes are involved in the complex network of cell-cell and cell-matrix interactions that dictate tumor cell behavior. The extensive remodeling of tumor microenvironment during cancer development and progression is associated with changes in the expression levels of HSPGs as well as in structural and functional alterations of HSPGs that affect cancer cell phenotype. Advances in understanding the molecular mechanisms underlying HSPG structural and functional variability in malignancy has provided promising HSPG-based therapeutic approaches for cancer treatment. HSPG targeting-based tumor treatment may involve the use of: (i) antibodies targeting selected HSPG epitopes or synthetic molecules that interfere with the functional binding of HSPGs with ligands such as growth factors or integrins and other receptors, thus affecting the downstream signaling and the related cellular processes such as adhesion, proliferation, migration, and invasion; (ii) small molecules that interfere with EV-mediated intercellular transfer of signaling molecules regulated by HSPGs; (iii) specific inhibitors or proteinase inhibitors that prevent HSPG shedding; (iv) drugs that regulate the expression levels of HSPGs in tumor microenvironment. However, as the knowledge on the multifaceted roles of HSPGs in tumor microenvironment progresses, innovative HSPG structure/function targeting strategies are explored to fight cancer.

## Figures and Tables

**Table 1:** **Heparan sulfate proteoglycan** (HSPG) nomenclature, human genes, schematic structure, cellular localization.

HSPG	Encoding Gene	Schematic Structure	Cellular Localization
Syndecan-1	SDC1	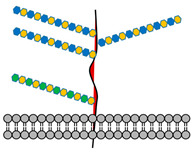	Cell surface
Syndecan-2	SDC2		
Syndecan-3	SDC3		
Syndecan-4	SDC4		
Glypican-1	GPC1	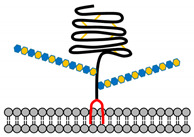	
Glypican-2	GPC2		
Glypican-3	GPC3		
Glypican-4	GPC4		
Glypican-5	GPC5		
Glypican-6	GPC6		
Perlecan	PRCAN	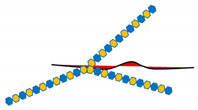	ECM, Basement membrane
Agrin	AGRN	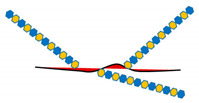	
Collagen type VIII	COL8A1	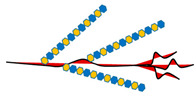	

**Table 2:** HS biosynthetic and modifying enzymes involved in cancer development and progression.

Enzyme	Gene	Type(s) of Cancer	Reference(s)
Xylosyltransferase1/2 (XYLT1/2)	*XYLT1-2*	Breast cancer/bone metastasis Salivary gland tumors	[64] [65]
β-1,4-Galactosyltransferase (b4Gal-T1-7)	*B4GALT1-7*	Breast cancer Colon cancer Liver cancer Leukemia Lung cancer Neuroblastoma Renal carcinoma	[66] [67] [68] [69] [70] [71] [72]
β-1,3-Glucuronyltransferase3 (GlcAT-I)	*B3GAT3*	Liver cancer	[73]
Exostosin like glycosyltransferase (EXTL1-3)	*EXTL1-3*	Breast cancer Hepatocarcinoma	[55] [74]
Exostosin1/2 (EXT1/2)	*EXT1-2*	Breast cancer Chondrosarcoma Osteochondroma Hepatocarcinoma Glioma Leukemia Thyroid tumor	[54,55] [75,76] [53,75,76] [77] [52] [57,58] [56]
N-deacetylase/N-sulfotransferase (1-4) (NDST1-4)	*NDST1-4*	Colorectal cancer Melanoma	[31,46] [78]
Glucuronyl C5-epimerase (GLCE)	*GLCE*	Breast cancer Lung cancer Prostate cancer	[59,60] [61] [62,63]
Hexuronyl 2-O-sulfotransferase (2-OST)	*HS2ST*	Breast cancer Multiple myeloma Prostate cancer	[79] [30] [49]
Glucosaminyl 6-O-sulfotransferase (6-OST)	*HS6ST*	Colorectal cancer Gastric cancer Glioma Ovarian cancer Pancreatic cancer	[50] [51] [52] [80,81] [82]
Glucosaminyl 3-O-sulfotransferase (3-OST)	*HS3ST*	Breast cancer Chondrosarcoma Colorectal cancer Leukemia Lung cancer Pancreatic cancer	[83] [47,83] [84] [85] [48] [86]
Endo-6-O-sulfatase1/2 (SULF1/2)	*SULF1-2*	Breast cancer Cervical cancer Liver tumors Ovarian cancer Other cancers	[87] [88] [89] [87] [90,91]
Heparanase (HPSE1/2)	*HPSE1-2*	Bladder cancer Brain tumors Breast cancer Gastric cancer Head and neck cancers Hepatocarcinoma Mesothelioma Myeloma Ovarian cancer Pancreatic cancer Sarcoma	[92] [93] [94,95] [96] [97] [98] [99] [100,101] [102] [103] [104]

**Table 3:** Differential expression of individual HSPGs in cancer.

HSPG	Changes in Expression Levels	Type(s) of Cancer	Reference(s)
SDC1	Increased	Bladder cancer, breast cancer, colorectal cancer, multiple myeloma, ovarian cancer, pancreatic ductal adenocarcinoma, squamous cell carcinoma	[29,31,35,105,108,109]
	Reduced	Cancer stem cell, colorectal cancer, endometrial cancer, hepatocellular carcinoma, mesothelioma, non-small-cell lung cancer, prostate cancer, sarcoma	[35,108,110,111]
SDC2	Increased	Bladder cancer, breast cancer, colorectal cancer, glioma, lung cancer, melanoma, prostate cancer	[112,113]
	Reduced	Osteosarcoma	[114]
SDC3	Increased	Bladder cancer, ovarian cancer, renal cell carcinoma	[115,116,117]
	Reduced	Neuroblastoma	[35]
SDC4	Increased	Ovarian cancer, papillary thyroid carcinoma	[115,118]
	Reduced	Neuroblastoma	[35]
GPC1	Increased	Breast cancer, esophageal squamous cell carcinoma, glioma, pancreatic cancer	[119,120,121,122,123]
	Reduced	Colorectal cancer, neuroblastoma	[35,105]
GPC2	Increased	Neuroblastoma, medulloblastoma, retinoblastoma	[124,125]
GPC3	Increased	Liver cancer, lung squamous cell carcinoma, neuroblastoma, ovarian cancer, testicular germ cell tumor, thyroid cancer, yolk sac tumor	[125,126,127,128,129]
	Reduced	Breast cancer, colorectal cancer, mesothelioma, non-small-cell lung cancer, neuroblastoma, renal cell carcinoma	[35,105,125,130]
GPC4	Increased	Colorectal cancer, pancreatic cancer	[31,131]
	Reduced	Breast cancer, ovarian carcinoma	[125,132,133]
GPC5	Increased	Rhabdomyosarcoma	[35,134,135]
	Reduced	Breast cancer, glioma, hepatocellular carcinoma, lung cancer, pancreatic cancer, prostate cancer	[136,137,138]
GPC6	Increased	Gastric cancer, melanoma	[139,140]
	Reduced	Colorectal cancer, ovarian cancer, retinoblastoma	[105,141,142]
Perlecan	Increased	Hepatocellular carcinoma, melanoma, pancreatic cancer, prostate cancer	[35,38,143,144,145,146]
	Reduced	Breast cancer, colorectal cancer, lung cancer, ovarian cancer, fibrosarcoma	[35,38,105,143,144,147]
Agrin	Increased	Cholangiocarcinoma, glioma, hepatocellular carcinoma, lung cancer, oral squamous cell carcinoma, rectal cancer	[38,148,149,150,151,152]
Collagen type VIII	Increased	Breast cancer, lung cancer, melanoma, ovary, pancreatic cancer, prostate cancer	[35,38,153]
	Reduced	Colorectal cancer	[105]

**Table 4:** Selected examples of HSPG targeting-based therapeutics for cancers.

Type of Drug	Target	Type(s) of Cancer	Reference(s)
Anti-GPC1 monoclonal antibody	Glypican-1	Esophageal squamous cell carcinoma	[244]
Monoclonal antibody HS20	Glypican-3 HS chain	Hepatocellular carcinoma	[245,246]
Human single-domain antibody specific for GPC2	Glypican-2	Neuroblastoma	[247]
Human recombinant antibody OC-46F2	Syndecan-1 ectodomain	Melanoma Ovarian carcinoma	[193] [248]
Antibody-pyrrolobenzodiazepine conjugate	Glypican-2	Neuroblastoma	[249]
Antibody-auristatin F conjugate	Glypican-1	Uterine cervical squamous cell carcinoma	[250]
HS mimetics G2.2	HSPG induced MAPK activation	Colon cancer stem cells	[251,252]
HS mimetics OTR4120 and OTR4131	HSPGs-mediated RANTES signaling	Hepatocellular carcinoma	[253]
Peptidic HS mimetics Synstatin	Syndecan-1/integrin/IGF1 complex formation	Mammary tumors Hepatocellular carcinoma	[210,226] [254]
Xylosides	HSPG biosynthesis	Glioma Lung cancer	[165,255,256] [257]
HS mimetics RK-682	Heparanase	Bladder cancer	[92,258,259]
HS mimetics PG545 (Pixatimod)	Heparanase	Mesothelioma Lymphoma Breast cancer	[260] [261] [262]
HS mimetics SST0001 (Roneparstat)	Heparanase	Sarcoma Myeloma	[263,264] [101]
HS mimetics M402 (Necuparanib)	Heparanase	Pancreatic cancer	[251,263,265]
HS mimetics PI-88 (Mupafostat)	Heparanase and Endoglucosamine 6-sulfatase	Hepatocellular carcinoma	[251,263]
Monoclonal antibodies 9E8 and H1023	Heparanase	Lymphoma Myeloma	[266] [266]
Triazolo-thiadiazoles	Heparanase	Hepatocellular carcinoma Lung cancer	[267] [267]
Phenyl sulfonyl compound OKN-007	Sulfatase 2	Hepatocellular carcinoma Glioblastoma	[268] [269]
Proteasome inhibitor (Bortezomib)	Sulfatase 2	Breast cancer	[270]

## Abbreviations

ADAM	a disintegrin and MMP protease
ADAMTS	ADAMS with thrombospondin motifs
ADC	antibody-drug-conjugates
AKT	protein kinase B
ECM	extracellular matrix
EGF	epidermal growth factor
ERK	extracellular signal-regulated kinase
EXT	Exostosin
EXTL	N-acetylglucosaminyltransferase
EV	extracellular vesicle
FAK	focal adhesion kinase
FGF	fibroblast growth factor
FGFR	fibroblast growth factor receptor
GAG	glycosaminoglycan
GalT	galactosyltransferase
GlcAT	glucuronyltransferase
GLCE	D-glucuronyl C5-epimerase
GPC	glypican
GPI	glycosylphosphatidylinositol
HB-EGF	heparin-binding epidermal growth factor-like
HGF	hepatocyte growth factor
HPSE	heparanase
HS	heparan sulfate
HSPG	heparan sulfate proteoglycan
HS3ST2	heparan sulfate glucosamine 3-O-sulfotransferase-2
HS6ST2	heparan sulfate glucosamine 6-O-sulfotransferase-2
IGF1	insulin-like growth factor-1
JAK	Janus kinase
MAPK	mitogen-activated protein kinase
MMP	matrix metalloproteinase
NDST	N-deacetylase/N-sulfotransferase
OST	heparan sulfate-O-sulfotransferase
PI3K	phosphatidylinositol 3-kinase
PKC	protein kinase C
PDGF	platelet-derived growth factor
PG	proteoglycans
SDC	syndecan
STAT	signal transducer and activator of transcription protein
SULF	endo-6-O-sulfatase
TGF	transforming growth factor
XYLT	xylosyltransferase
VEGF	vascular endothelial growth factor
